# Diet Quality and Biological Risk in a National Sample of Older Americans

**DOI:** 10.1093/geroni/igaa057.3378

**Published:** 2020-12-16

**Authors:** Yeon Jin Choi, Jennifer Ailshire, Jung Ki Kim, Eileen Crimmins

**Affiliations:** University of Southern California, Los Angeles, California, United States

## Abstract

Biomarkers are sensitive to current health status and capture aspects of health that may precede the development of disease and other health problems. Using comprehensive measures of biological risk, this study aims to investigate the relationship between intake of individual dietary components, overall diet quality, and biological dysregulation. For the analysis, we used nutrition and biomarker data from 3,641 older adults (over age 50) in the Health and Retirement Study. Eleven out of 13 individual dietary components were associated with lower biological risk. After controlling for SES, health behaviors, and access to health care, a high intake of fruits, greens and beans, whole grains, seafood and plant proteins, and fatty acids and a low intake of sodium and saturated fat were still associated with lower biological risk. Respondents with poor/suboptimal quality diet had higher biological risk than those with good quality diet. After controlling for SES, health behaviors, and access to health care, respondents with poor/suboptimal quality diet continued to exhibit higher biological risk than those with good quality diet, though the differences in biological risk were reduced. Findings from this study emphasize the importance of healthy eating in improving health of older adults. Encouraging intake of fruits, greens and beans, whole grains, seafood and plant proteins, and fatty acids, while limiting consumption of sodium and saturated fat would improve overall diet quality and contribute to the prevention of chronic diseases and morbidity.

